# Finite-Time Asynchronous Event-Triggered Formation of UAVs with Semi-Markov-Type Topologies

**DOI:** 10.3390/s22124529

**Published:** 2022-06-15

**Authors:** Chao Ma, Suiwu Zheng, Tao Xu, Yidao Ji

**Affiliations:** 1 School of Mechanical Engineering, University of Science and Technology Beijing, Beijing 100083, China; cma@ustb.edu.cn (C.M.); yidao.ji@xs.ustb.edu.cn (Y.J.); 2 State Key Lab of Management and Control for Complex Systems, Institute of Automation, Chinese Academy of Sciences, Beijing 100190, China; xutao2020@ia.ac.cn; 3 University of Chinese Academy of Sciences, Beijing 100190, China

**Keywords:** finite-time formation, event-triggered formation, UAVs, semi-Markov topologies

## Abstract

In this paper, the finite-time formation problem of UAVs is investigated with consideration of semi-Markov-type switching topologies. More precisely, finite-time passivity performance is adopted to overcome the dynamical effect of disturbances. Furthermore, an asynchronous event-triggered communication scheme is proposed for more efficient information exchanges. The mode-dependent formation controllers are designed in terms of the Lyapunov–Krasovskii method, such that the configuration formation can be accomplished. Finally, simulation results are given to demonstrate the validity of the proposed formation approach.

## 1. Introduction

With the rapid development of control and network technology, unmanned aerial vehicles (UAV) have been a prominent research topic in both academic and engineering fields in recent years. A variety of application scenarios have been employed by UAVs, such as search and rescue [1,2,3], express transportation [4,5], and remote sensing [6]. Furthermore, it is worth mentioning that multiple UAVs can accomplish complex tasks with more robustness and efficient working ability than a single UAV. As one of the interesting control issues, the formation problem for UAVs has gained significant research attention [7,8,9,10]. Specifically, the communication topology for UAVs has played a key role since all the information exchanges are achieved via the communication network. It should be pointed out that it is always difficult or expensive to hold fixed communication topology within different task assignments or in an unstructured environment with disturbances. Furthermore, the varying topology may exhibit certain random features in some conditions. Hence, it is natural and important to investigate the formation protocol with random topologies [11,12,13]. On the other hand, lots of studies have shown that the Markov process could be adopted to describe these stochastic jumping features between different modes accurately. As a result, some initial efforts have been made toward Markovian jumping communication topologies. However, notice that the transition probabilities of a Markovian jumping system are conformed to be a fixed exponential distribution, which would lead to certain restrictions in practical applications. In fact, time-varying transition probabilities are more general in implementations, and the semi-Markov process is able to depict these time-varying transition probabilities [14,15,16,17]. As far as the authors’ knowledge extends, there is still huge room for studies on the integration of semi-Markovian jumping topology and UAV formation problems.

Another active research field is networked control systems, where the event-triggered schemes have aroused great concern recently [18,19,20]. As we well know, computational and network resources are very valuable for UAVs. Under this context, the event-triggered communication schemes could considerably improve communication efficiency and optimize data transmission compared to traditional time-triggered ones [21,22,23]. Moreover, when taking into account the topology jumpings, the vast majority of event-triggered schemes do not utilize mode information well, and thus, the acquired results are conservative in certain senses. It is noteworthy that control strategies for Markov/semi-Markov jumping systems could utilize the involved information on active modes to improve control effects substantially. In short, the mode-dependent event-triggered strategies are more applicable in practical applications [14,24]. However, it is necessary to point out the fact that constructing an available mode-dependent triggering scheme by events is difficult and challenging. To date, to the best of our knowledge, there are few results on the formation of UAVs with semi-Markovian jumping topology. This motivates us to shorten this gap.

In light of the aforementioned discussions, our aim is to explore the mode-dependent event-triggered strategy for UAVs formation problems with semi-Markovian jumping topology within a finite-time framework. More precisely, the concept of finite-time passivity is employed for disturbance attenuation during UAV formation while the transient performance of formation dynamics is achieved. Compared with most reporting works, the novelties of our paper are threefold. First, the finite-time formation model of UAVs is formulated with sufficient consideration of semi-Markovian jumping topologies. Secondly, an original asynchronous mode-dependent event-triggering strategy is developed for UAVs in order to improve the communication efficiency through reducing data transmissions. Thirdly, according to model transformation and the Lyapunov–Krasovskii functional, a distributed formation protocol has been developed to satisfy the finite-time passivity performance in the mean-square sense.

The rest of our work is as listed following. In Section 2, we construct the formation model of UAVs with regard to semi-Markovian jumping topologies and design the asynchronous mode-dependent event-triggering formation protocol. Then, sufficient conditions satisfying finite-time passivity performance would be verified and given with reliable details in Section 3. After that, the simulation results are illustrated with numerical simulations in Section 4 to verify the correctness of our derived designs. Finally, the conclusion and expectation of this paper are given in Section 5.

The notations in this paper are given in Table 1.

## 2. Problem Formulation

Given a probability space (O,F,P) and denote {δ(t),t≥0} as a continuous-time discrete-state semi-Markov process, which takes values in a finite set S={1,…,N}. The transition probability matrix $\Pi :=(\pi_{ij}\left(h\right))$, h>0, ∀i,j∈S is defined as follows

$$\begin{array}{cc} \; & \mathrm{Pr}(\delta (t+h)=j|\delta (t)=i)\\ \; & =\left\{\begin{array}{c} \pi_{ij}\left(h\right)h+o\left(h\right),\;\;i\neq j,\\ 1+\pi_{ii}\left(h\right)h+o\left(h\right),\;\;i=j, \end{array}\right. \end{array}$$

$$\begin{array}{cc} \; & \pi_{ij}\left(h\right)\geq 0,i\neq j,\\ \; & \pi_{ii}\left(h\right)=-\sum_{j=1,\;j\neq i}^{N}\pi_{ij}\left(h\right),\forall i\in S. \end{array}$$

Accordingly, we introduce a directed graph $G_{\delta (t)}=\left\{V_{\delta (t)},E_{\delta (t)},A_{\delta (t)}\right\}$ to depict the communication topology among the UAVs. $V_{\delta (t)}=\left\{v_{1\delta (t)},\cdots ,v_{\delta (t)}\right\}$ and $E_{\delta (t)}$ represent for the sets of nodes and edges, $A_{\delta (t)}={\left[a_{ij\delta (t)}\right]}_{N\times N}\in R^{N\times N}$ represents the weighted adjacency matrix by

(1) $$\left\{\begin{array}{c} a_{ij\delta (t)}>0,\left(v_{i\delta (t)},v_{j\delta (t)}\right)\in E_{\delta (t)},\\ a_{ij\delta (t)}=0,\mathrm{Otherwise}. \end{array}\right.$$

respectively. Moreover, the Laplacian matrix of $G_{\delta (t)}$ is defined by $L_{\delta (t)}={\left[l_{ij\delta (t)}\right]}_{N\times N}\in R^{N\times N}$ with

(2) $$\left\{\begin{array}{c} l_{ij\delta (t)}=-a_{ij\delta (t)},i\neq j\\ l_{ii\delta (t)}=\sum_{j=1,i\neq j}^{N}a_{ij\delta (t)}. \end{array}\right.$$

By employing the graph theory, one can verify for any possible $L_{\delta (t)}$ and a full row rank matrix $E\in R^{(N-1)\times N}$ [25]

$$E=\left[\begin{array}{ccccc} 1 & -1 & 0 & \cdots & 0\\ 0 & 1 & -1 & \cdots & 0\\ \vdots & \vdots & \ddots & \vdots & \vdots \\ 0 & 0 & 0 & 1 & -1 \end{array}\right],$$

there must exist a matrix $M_{\delta (t)}\in R^{N\times (N-1)}$ satisfying $L_{\delta (t)}=M_{\delta (t)}E$.

For the formation of UAVs, a typical outer/inner-loop design structure is utilized [26]. Then, consider the group of *N* quadrotors with double integrator dynamics described as follows:

$$\begin{array}{cc} \; & \left\{\begin{array}{c} {\dot{p}}_{m}\left(t\right)=v_{m}\left(t\right)\\ {\dot{v}}_{m}\left(t\right)=u_{m}\left(t\right) \end{array},\right.\\ \; & m=1,2,\ldots ,N, \end{array}$$

where $p_{m}\in R^{n}$, $v_{m}\in R^{n}$ and $u_{m}\in R^{n}$ represent the position, velocity and formation control input vector, respectively.

**Remark** **1.** *The notable outer/inner-loop formation control configuration for quadrotors has been widely studied, where the outer-loop control is devoted to the desired position and velocity of UAVs. Under this context, the double integrator point-mass model can be effectively adopted to describe the formation dynamics of UAVs.*

By taking into account the external disturbances, the following state-space model of each UAV can be rewritten by:

(3) $${\dot{x}}_{m}\left(t\right)=Ax_{m}\left(t\right)+Bu_{m}\left(t\right)+Bw\left(t\right),$$

where $x_{m}={\left(p_{m}^{T},v_{m}^{T}\right)}^{T}$, $u_{m}\left(t\right)$ denotes the formation control input, w(t) represents the external disturbances and

$$\begin{array}{cc} A= & \left[\begin{array}{cc} 0 & I\\ 0 & 0 \end{array}\right],\\ B= & \left[\begin{array}{c} 0\\ I \end{array}\right]. \end{array}$$

Suppose that all UAVs share a unified communication network with semi-Markovian jumping topologies. Accordingly, we assume that a mode-dependent sampling period $h_{\delta (t)},$$h_{\delta (t)}\leq \bar{h},$ is adopted, such that local information exchanges are accomplished between neighboring UAVs. Furthermore, the following asynchronous mode-dependent event triggering communication strategy is applied

$$\begin{array}{cc} t_{k+1}^{m}h_{\delta (t)}= & t_{k}^{m}h_{\delta (t)}+\min_{l_{m}\geq 1}\left\{l_{m}h_{\delta (t)}\right|\chi_{m}^{T}\left(t_{k}^{m}h_{\delta (t)}+l_{m}h_{\delta (t)}\right)W_{1\delta (t)}\chi_{m}\left(t_{\sigma }^{m}h_{\delta (t)}+l_{m}h_{\delta (t)}\right)\\ \geq & \kappa_{\delta (t)}\varkappa_{m}^{T}\left(t_{k}^{m}h_{\delta (t)}+l_{m}h_{\delta (t)}\right)W_{2\delta (t)}\varkappa_{m}\left(t_{k}^{m}h_{\delta (t)}+l_{m}h_{\delta (t)}\right)\}, \end{array}$$

where

$$\begin{array}{cc} \; & \chi_{m}\left(t_{k}^{m}h_{\delta (t)}+l_{m}h_{\delta (t)}\right)\\ = & x_{m}\left(t_{k}^{m}h_{\delta (t)}+l_{m}h_{\delta (t)}\right)-x_{m}\left(t_{k}^{m}h_{\delta (t)}\right),\\ \; & \varkappa_{m}\left(t_{k}^{m}h_{\delta (t)}+l_{m}h_{\delta (t)}\right)\\ = & \sum_{n=1}^{N}a_{mn\delta (t)}\left(x_{m}\left(t_{k}^{m}h_{\delta (t)}\right)-x_{n}\left(t_{k^{*}}^{m}h_{\delta (t)}\right)\right),\\ \; & k^{*}=\arg\min_{\varrho }\left\{t_{k}^{m}+l_{m}-t_{\varrho }^{n}|t_{k}^{m}+l_{m}>t_{\varrho }^{n},\varrho \in N\right\}, \end{array}$$

and $t_{k}^{m}$ represents the latest *k*th updating instant of the *m*th UAV, $0<\kappa_{\delta (t)}<1$ represents the triggering threshold, $W_{1\delta (t)}>0$ and $W_{2\delta (t)}>0$ represents the weighting matrices. It can be found that based on the proposed asynchronous event-triggered strategy, the information updates of the UAVs only require the neighboring information exchanges instead of the overall states of UAVs, which is more applicable for the distributed local communications of the UAVs. In conclusion, detailed notations of these parameters are given in the following Table 2.

In the sequel, the formation configuration definition is given as follows:

**Definition** **1.** *The distributed formation configuration can be accomplished if it holds that*

$$\lim_{t\to \infty }∥x_{m}-x_{n}-d_{mn}∥=0,$$

*where $d_{mn}$ represents the relative formation configuration distance.*

Denote $d_{mn}=d_{m}-d_{n}$, and design the formation control input by

$$\begin{array}{cc} u_{m}\left(t\right)= & K_{\delta (t)}\sum_{m=1}^{N}a_{mn\delta (t)}(x_{m}\left(t_{k}^{m}h_{\delta (t)}\right)\\ \; & -x_{n}\left(t_{k^{*}}^{m}h_{\delta (t)}\right)-d_{mn}),t\in \left[t_{k}^{m}h_{\delta (t)},t_{k^{*}}^{m}h_{\delta (t)}\right), \end{array}$$

where $K_{\delta (t)}$ represents the mode-dependent gains to be determined. As a result, by letting $\zeta_{m}=x_{m}-d_{m}$ and dividing $t_{k}^{m}\leq l<t_{k+1}^{m}$ into $t_{k+1}^{m}-t_{k}^{m}$ intervals, it yields that

$$\begin{array}{cc} {\dot{\zeta }}_{m}\left(t\right)= & A\zeta_{m}\left(t\right)+BK_{\delta (t)}\sum_{m=1}^{N}a_{mn\delta (t)}(\zeta_{m}\left(t_{k}^{m}h_{\delta (t)}\right)\\ \; & -\zeta_{n}\left(t_{k^{*}}^{m}h_{\delta (t)}\right))+Bw\left(t\right),t\in \left[t_{k}^{m}h_{\delta (t)},t_{k^{*}}^{m}h_{\delta (t)}\right). \end{array}$$

Based on the above results, one can further obtain that

$$\begin{array}{cc} {\dot{\zeta }}_{m}\left(t\right)= & A\zeta_{m}\left(t\right)+BK_{\delta (t)}\sum_{m=1}^{N}a_{mn\delta (t)}(\zeta_{m}\left(lh_{\delta (t)}\right)\\ \; & -\zeta_{n}\left(lh_{\delta (t)}\right)-e_{m}\left(lh_{\delta (t)}\right)+e_{n}\left(lh_{\delta (t)}\right))+Bw\left(t\right),\\ \; & t\in [lh_{\delta (t)},\left(l+1\right)h_{\delta (t)}). \end{array}$$

where

$$e_{m}\left(lh_{\delta (t)}\right)=\zeta_{m}\left(lh_{\delta (t)}\right)-\zeta_{m}\left(t_{k}^{m}h_{\delta (t)}\right).$$

Note that the resulting closed-loop dynamics can be rewritten as follows:

$$\begin{array}{cc} \dot{\zeta }\left(t\right)= & \left(I_{N}⊗A\right)\zeta \left(t\right)+\left(L_{\delta (t)}⊗BK_{\delta (t)}\right)\times \\ \; & \left(\zeta \left(lh_{\delta (t)}\right)-e\left(lh_{\delta (t)}\right)\right)+\left(I⊗B\right)w\left(t\right), \end{array}$$

where

$$\begin{array}{cc} \zeta (t)= & {\left[\zeta_{1}^{T}\left(t\right),\zeta_{2}^{T}\left(t\right),\ldots ,\zeta_{N}^{T}\left(t\right)\right]}^{T},\\ e(t)= & {\left[e_{1}^{T}\left(t\right),e_{2}^{T}\left(t\right),\ldots ,e_{N}^{T}\left(t\right)\right]}^{T}. \end{array}$$

For simplicity of description, denote δ(t)=i and one has

$$\begin{array}{cc} \dot{\zeta }\left(t\right)= & \left(I_{N}⊗A\right)\zeta \left(t\right)+\left(L_{i}⊗BK_{i}\right)\times \\ \; & \left(\zeta \left(lh_{i}\right)-e\left(lh_{i}\right)\right)+\left(I⊗B\right)w\left(t\right). \end{array}$$

Recalling that $L_{\delta (t)}=M_{\delta (t)}E$, it can be obtained that

$$\begin{array}{cc} \dot{\eta }\left(t\right)= & \left(I_{N-1}⊗A\right)\eta \left(t\right)+\left({\mathrm{EM}}_{i}⊗BK_{i}\right)\times \\ \; & \left(\eta \left(lh_{i}\right)-\epsilon \left(lh_{i}\right)\right)+\left(I_{N-1}⊗B\right)\omega \left(t\right), \end{array}$$

where η(t)=(E⊗I)ζ(t) represents the formation error of ζ(t) and ϵ(t)=(E⊗I)e(t), ω(t)=(E⊗I)w(t), respectively.

Before proceeding, the following definitions are given for the transient performance of formation dynamics [27,28].

**Definition** **2.** *Given constants $c_{1}>0$, $c_{2}>0$, $\bar{\omega }>0$, $T_{F}>0$ and matrix Λ>0, the finite-time boundedness formation is achieved with respect to $\left(c_{1},c_{2},\bar{\omega },T_{F}\right)$ in a mean-square sense, if it holds that*

$$\begin{array}{cc} \; & \left\{\begin{array}{c} E\left\{\eta^{T}\left(t_{0}\right)\Lambda \eta \left(t_{0}\right)\right\}\leq c_{1}\\ \int_{0}^{T_{F}}\omega^{T}\left(s\right)\omega \left(s\right)ds\leq \bar{\omega } \end{array}\right.⟹E\left\{\eta^{T}\left(t\right)\Lambda \eta \left(t\right)\right\}\leq c_{2},\\ \; & c_{2}>c_{1},t\in \left[0,T_{F}\right]. \end{array}$$

**Definition** **3.** *Given constants $c_{1}>0$, $c_{2}>0$, $\bar{\omega }>0$, γ>0, $T_{F}>0$ and matrix Λ>0, the finite-time passivity formation is achieved with respect to $\left(c_{1},c_{2},\bar{\omega },\gamma ,T_{F}\right)$ in mean-square sense, if finite-time boundedness is satisfied and it holds that*

$$\begin{array}{c} 2E\left\{\int_{0}^{T_{F}}\eta^{T}\left(s\right)\omega \left(s\right)ds\right\}+\gamma \int_{0}^{T_{F}}\omega^{T}\left(s\right)\omega \left(s\right)ds\geq 0. \end{array}$$

**Remark** **2.** *It should be pointed out that based on model transformation, the finite-time formation control problem is converted to the finite-time stability (passivity performance) control problem. The concept of finite-time stability (passivity performance) of formation errors is then investigated, where the asymptotic behavior over a finite-time interval is concerned instead of infinite-time stability, which implies that the prescribed upper bound can be satisfied during the prescribed time intervals. The mean-square finite-time passivity performance not only focuses on the dynamical features within a finite time interval but also deals with the disturbances from an energy point of view. It should be pointed out that mean-square finite-time passivity may not be Lyapunov mean-square passivity and vice versa.*

Hence, our main aim is to design the mode-dependent formation control gains $K_{i}$ to guarantee finite-time passivity formation. The following matrix lemma can be provided to derive further results [29].

**Lemma** **1.** *Given matrix Φ>0, τ(t) satisfying $0\leq \tau \left(t\right)\leq \bar{\tau }$, $\bar{\tau }>0$, and $\dot{x}\left(t\right):\left[-\bar{\tau },0\right]\to R^{n}$ such that it holds that*

$$-\bar{\tau }\int_{t-\bar{\tau }}^{t}{\dot{x}}^{T}\left(s\right)\Phi \dot{x}\left(s\right)ds\leq \chi^{T}\left(t\right)\Psi \chi \left(t\right),$$

*where*

$$\begin{array}{cc} \chi (t)= & {\left[x^{T}\left(t\right),x^{T}\left(t-\tau \left(t\right)\right),x^{T}\left(t-\bar{\tau }\right)\right]}^{T},\\ \Psi = & \left[\begin{array}{ccc} -\Phi & \Phi & 0\\ {}* & -2\Phi & \Phi \\ {}* & * & -\Phi \end{array}\right]. \end{array}$$

## 3. Main Results

This section gives the detailed formation controller design procedure with the aid of convex optimization and the matrix technique.

**Theorem** **1.** *For given $\bar{h}$, the finite-time formation problem of UAVs (3) can be solved with designed formation controller gains if there exist mode-dependent matrices P(i)≻0, Q≻0, R≻0, such that $\Xi_{i}\left(h\right)≺0$ holds for all i∈S, where*

$$\begin{array}{cc} \Xi_{i}\left(h\right)= & \left[\begin{array}{cc} \Xi_{1i}\left(h\right) & \Xi_{2i}\left(h\right)\\ {}* & \Xi_{3i}\left(h\right) \end{array}\right],\\ \Xi_{1i}\left(h\right)= & \left[\begin{array}{cc} \Xi_{11i}\left(h\right) & \left({\mathrm{EM}}_{i}⊗P_{i}BK_{i}\right)+\left(I⊗R\right)\\ {}* & -2\left(I⊗R\right)+\kappa_{i}\left(EE^{T}⊗W_{2i}\right) \end{array}\right],\\ \Xi_{11i}\left(h\right)= & 2\left(I⊗P_{i}A\right)+\left(I⊗Q\right)-\left(I⊗R\right)\\ \; & -\alpha \left(I⊗P_{i}\right)+\sum_{j=1}^{N}\pi_{ij}\left(h\right)\left(I⊗P_{j}\right),\\ \Xi_{2i}\left(h\right)= & \left[\Xi_{21i}\left(h\right),\Xi_{22i}\left(h\right)\right], \end{array}$$

$$\begin{array}{cc} \Xi_{21i}\left(h\right)= & \left[\begin{array}{cc} 0 & -({\mathrm{EM}}_{i}⊗P_{i}BK_{i})\\ \left(I⊗R\right) & 0 \end{array}\right],\\ \Xi_{22i}\left(h\right)= & \left[\begin{array}{cc} I⊗P_{i}B_{w} & \bar{h}{\left(I⊗A_{i}\right)}^{T}\\ 0 & \bar{h}{\left({\mathrm{EM}}_{i}⊗BK_{i}\right)}^{T} \end{array}\right],\\ \Xi_{3i}\left(h\right)= & \left[\begin{array}{cc} \Xi_{31i}\left(h\right) & \Xi_{32i}\left(h\right)\\ {}* & \Xi_{33i}\left(h\right) \end{array}\right],\\ \Xi_{31i}\left(h\right)= & \left[\begin{array}{cc} -(I⊗Q)-(I⊗R) & 0\\ {}* & -(I⊗W_{1i}) \end{array}\right],\\ \Xi_{32i}\left(h\right)= & \left[\begin{array}{cc} 0 & 0\\ 0 & -\bar{h}{\left({\mathrm{EM}}_{i}⊗BK_{i}\right)}^{T} \end{array}\right],\\ \Xi_{33i}\left(h\right)= & \left[\begin{array}{cc} -\alpha I & \bar{h}{\left(I⊗B_{w}\right)}^{T}\\ {}* & -{\left(I⊗R\right)}^{-1} \end{array}\right]. \end{array}$$

*and*

$$c_{1}\phi_{1}+\bar{h}c_{1}\phi_{2}+\frac{1}{2}{\bar{h}}^{3}c_{1}\phi_{3}+\bar{\omega }\left(1-e^{-aT_{F}}\right)\leq \phi_{4}e^{-aT_{F}}c_{2},$$

*with $\phi_{1}=\max\left\{\lambda_{\max}\left\{P_{i}\right\}\right\},\phi_{2}=\lambda_{\max}\left\{Q\right\},\phi_{3}=\lambda_{\max}\left\{R\right\},\phi_{4}=\min\left\{\lambda_{\min}\left\{P_{i}\right\}\right\}.$*

**Proof.**  Construct mode-dependent Lyapunov–Krasovskii functionals as follows:

(4) $$V\left(i,t\right)=V_{1}\left(i,t\right)+V_{2}\left(i,t\right)+V_{3}\left(i,t\right),i\in S,$$

where

$$\begin{array}{cc} \; & V_{1}\left(i,t\right)=\eta^{T}\left(t\right)\left(I⊗P_{i}\right)\eta \left(t\right),\\ \; & V_{2}\left(i,t\right)=\int_{t-\bar{h}}^{t}\eta^{T}\left(s\right)\left(I⊗Q\right)\eta \left(s\right)ds,\\ \; & V_{3}\left(i,t\right)=\bar{h}\int_{-\bar{h}}^{0}\int_{t+\varsigma }^{t}{\dot{\eta }}^{T}\left(s\right)\left(I⊗R\right)\dot{\eta }\left(s\right)dsd\varsigma . \end{array}$$

Moreover, we defined the weak infinitesimal operator for V(i,t) by

$$LV\left(i,t\right)=\lim_{\Delta \to 0}\frac{1}{\Delta }\left\{E\left\{V\left(\delta \left(t+\Delta \right),t+\Delta \right)|\delta \left(t\right)=i\right\}-V\left(i,t\right)\right\},$$

with

$$\begin{array}{cc} \; & \lim_{\Delta \to 0}\frac{1}{\Delta }\frac{\Gamma_{i}\left(h+\Delta \right)-\Gamma_{i}\left(h\right)}{1-\Gamma_{i}\left(h\right)}=0,\\ \; & \lim_{\Delta \to 0}\frac{1}{\Delta }\frac{1-\Gamma_{i}\left(h+\Delta \right)}{1-\Gamma_{i}\left(h\right)}=1,\\ \; & \lim_{\Delta \to 0}\frac{1}{\Delta }\frac{q_{ij}\left(\Gamma_{i}\left(h\right)-\Gamma_{i}\left(h+\Delta \right)\right)}{\Delta (1-\Gamma_{i}\left(h\right))}\\ \; & =q_{ij}\pi_{i}\left(h\right)=\pi_{ij}\left(h\right), \end{array}$$

where $\Gamma_{i}\left(h\right)$ represents the cumulative distribution function of the sojourn time, and $q_{ij}$ denotes the probability intensity.

By employing the input-delay strategy, one has

$$\begin{array}{cc} \dot{\eta }\left(t\right)= & \left(I⊗A_{i}\right)\eta \left(t\right)+\left({\mathrm{EM}}_{i}⊗BK_{i}\right)\times \\ \; & \left(\eta \left(t-\tau_{i}\left(t\right)\right)\right)-\epsilon \left(lh_{i}\right))+\left(I⊗B_{w}\right)\omega \left(t\right), \end{array}$$

where $\tau_{i}\left(t\right)=t-lh_{i},t\in \left[lh_{i},\left(l+1\right)h_{i}\right)$ with $0\leq \tau_{i}\left(t\right)<\bar{h}.$ As a result, it can be derived that

$$\begin{array}{cc} LV_{1}\left(i,t\right)= & \lim_{▵\to 0}\frac{1}{▵}[\sum_{j=1,j\neq i}^{N}\mathrm{Pr}\left\{\sigma \left(t+▵\right)=j\right|\\ \; & \sigma \left(t\right)=i\}\eta^{T}\left(t+▵\right)P_{j}\eta \left(t+▵\right)\\ \; & +\mathrm{Pr}\left\{\sigma \left(t+▵\right)=i\left|\sigma (t)=i\right.\right\}\times \\ \; & \eta^{T}\left(t+▵\right)P_{i}\eta \left(t+▵\right)-\eta^{T}\left(t\right)P_{i}\eta \left(t\right)]\\ \; & \lim_{▵\to 0}\frac{1}{▵}[\sum_{j=1,j\neq i}^{N}\frac{q_{ij}\left(\Gamma_{i}\left(h+▵\right)-\Gamma_{i}\left(h\right)\right)}{1-\Gamma_{i}\left(h\right)}\times \\ \; & \eta^{T}\left(t+▵\right)P_{j}\eta \left(t+▵\right)\\ \; & +\frac{\left(\Gamma_{i}\left(h+▵\right)-\Gamma_{i}\left(h\right)\right)}{1-\Gamma_{i}\left(h\right)}\times \\ \; & \eta^{T}\left(t+▵\right)P_{i}\eta \left(t+▵\right)]-\eta^{T}\left(t\right)P_{i}\eta \left(t\right)\\ = & {\dot{\eta }}^{T}\left(t\right)P_{i}\eta \left(t\right)+\eta^{T}\left(t\right)P_{i}\dot{\eta }\left(t\right)\\ \; & +\sum_{j=1}^{N}\pi_{ij}\left(h\right)\eta^{T}\left(t\right)P_{j}\eta \left(t\right)\\ = & 2\eta^{T}\left(t\right)P_{i}\dot{\eta }\left(t\right)+\sum_{j=1}^{N}\pi_{ij}\left(h\right)\eta^{T}\left(t\right)P_{j}\eta \left(t\right)\\ = & 2\eta^{T}\left(t\right)P_{i}(\left(I_{N}⊗A_{i}\right)\eta \left(t\right)+\left({\mathrm{EM}}_{i}⊗BK_{i}\right)\times \\ \; & \left(\eta \left(t-\tau_{i}\left(t\right)\right)-\epsilon \left(lh_{i}\right)\right)+\left(I⊗B_{w}\right)\omega \left(t\right))\\ \; & +\sum_{j=1}^{N}\pi_{ij}\left(h\right)\eta^{T}\left(t\right)P_{j}\eta \left(t\right) \end{array}$$

Similarly, it can be obtained that

$$LV_{2}\left(i,t\right)=\eta^{T}\left(t\right)\left(I⊗Q\right)\eta \left(t\right)-\eta^{T}\left(t-\bar{h}\right)\left(I⊗Q\right)\eta \left(t-\bar{h}\right)$$

$$LV_{3}\left(i,t\right)={\bar{h}}^{2}{\dot{\eta }}^{T}\left(t\right)\left(I⊗R\right)\dot{\eta }\left(t\right)-\bar{h}\int_{\bar{h}}^{0}\dot{\eta }\left(s\right)\left(I⊗R\right)\dot{\eta }\left(s\right)ds.$$

By employing Lemma 1 to $LV_{3}\left(i,t\right)$, one has

$$\begin{array}{cc} LV_{3}\left(i,t\right)= & {\bar{h}}^{2}{\dot{\eta }}^{T}\left(t\right)\left(I⊗R\right)\dot{\eta }\left(t\right)\\ \; & -\bar{h}\int_{\bar{h}}^{0}\dot{\eta }\left(s\right)\left(I⊗R\right)\dot{\eta }\left(s\right)ds\\ \leq & \xi^{T}{\left[\begin{array}{c} {\left(I⊗A_{i}\right)}^{T}\\ {\left({\mathrm{EM}}_{i}⊗BK_{i}\right)}^{T}\\ 0\\ -{\left({\mathrm{EM}}_{i}⊗BK_{i}\right)}^{T}\\ {\left(I⊗B_{w}\right)}^{T} \end{array}\right]}^{T}\times \end{array}$$

$$\begin{array}{cc} \; & \left(I⊗R\right)\left[\begin{array}{c} {\left(I⊗A_{i}\right)}^{T}\\ {\left({\mathrm{EM}}_{i}⊗BK_{i}\right)}^{T}\\ 0\\ -{\left({\mathrm{EM}}_{i}⊗BK_{i}\right)}^{T}\\ {\left(I⊗B_{w}\right)}^{T} \end{array}\right]\xi , \end{array}$$

where $\xi^{T}={\left[\eta^{T}\left(t\right),\eta^{T}\left(t-\tau_{i}\left(t\right)\right),\zeta^{T}\left(t-\bar{h}\right),\epsilon^{T}\left(lh_{i}\right),\omega^{T}\left(t\right)\right]}^{T}.$

Meanwhile, the event-triggered function implies that

$$\begin{array}{cc} \; & -\epsilon^{T}\left(lh_{i}\right)\left(I⊗W_{1i}\right)\epsilon \left(lh_{i}\right)\\ \; & +\kappa_{i}\eta^{T}\left(t-\tau_{i}\left(t\right)\right)\left(EE^{T}⊗W_{2i}\right)\eta \left(t-\tau_{i}\left(t\right)\right)\geq 0. \end{array}$$

Then, we can derive the following matrix inequality

$$\begin{array}{cc} \; & LV\left(i,t\right)-\alpha \eta^{T}\left(t\right)\left(I⊗P_{i}\right)\eta \left(t\right)-\alpha \omega^{T}\left(t\right)\omega \left(t\right)\\ \leq & LV\left(i,t\right)-\alpha \eta^{T}\left(t\right)\left(I⊗P_{i}\right)\eta \left(t\right)-\alpha \omega^{T}\left(t\right)\omega \left(t\right)\\ \; & -\epsilon^{T}\left(lh_{i}\right)\left(I⊗W_{1i}\right)\epsilon \left(lh_{i}\right)+\kappa_{i}\eta^{T}\left(t-\tau_{i}\left(t\right)\right)\\ \; & \times \left(EE^{T}⊗W_{2i}\right)\eta \left(t-\tau_{i}\left(t\right)\right)\\ \leq & \xi^{T}\left({\tilde{\Xi }}_{i}\left(h\right)+{\bar{h}}^{2}{\left[\begin{array}{c} {\left(I⊗A_{i}\right)}^{T}\\ {\left({\mathrm{EM}}_{i}⊗BK_{i}\right)}^{T}\\ 0\\ -{\left({\mathrm{EM}}_{i}⊗BK_{i}\right)}^{T}\\ {\left(I⊗B_{w}\right)}^{T} \end{array}\right]}^{T}\times \right.\\ \; & \left.\left(I⊗R\right)\left[\begin{array}{c} {\left(I⊗A_{i}\right)}^{T}\\ {\left({\mathrm{EM}}_{i}⊗BK_{i}\right)}^{T}\\ 0\\ -{\left({\mathrm{EM}}_{i}⊗BK_{i}\right)}^{T}\\ {\left(I⊗B_{w}\right)}^{T} \end{array}\right]\right)\xi \end{array}$$

where

$$\begin{array}{cc} {\tilde{\Xi }}_{i}\left(h\right)= & \left[\begin{array}{cc} {\tilde{\Xi }}_{1i}\left(h\right) & {\tilde{\Xi }}_{2i}\left(h\right)\\ {}* & {\tilde{\Xi }}_{3i}\left(h\right) \end{array}\right],\\ {\tilde{\Xi }}_{1i}\left(h\right)= & \left[\begin{array}{cc} {\tilde{\Xi }}_{11i}\left(h\right) & \left({\mathrm{EM}}_{i}⊗P_{i}BK_{i}\right)+\left(I⊗R\right)\\ {}* & -2\left(I⊗R\right)+\kappa_{i}\left(E^{T}E⊗W_{2i}\right) \end{array}\right],\\ {\tilde{\Xi }}_{11i}\left(h\right)= & 2\left(I⊗P_{i}A\right)+\left(I⊗Q\right)-\left(I⊗R\right)\\ \; & -\alpha \left(I⊗P_{i}\right)+\sum_{j=1}^{N}\pi_{ij}\left(h\right)\left(I⊗P_{j}\right),\\ {\tilde{\Xi }}_{2i}\left(h\right)= & \left[\begin{array}{ccc} 0 & -({\mathrm{EM}}_{i}⊗P_{i}BK_{i}) & I⊗P_{i}B_{w}\\ \left(I⊗R\right) & 0 & 0 \end{array}\right],\\ {\tilde{\Xi }}_{3i}\left(h\right)= & \left[\begin{array}{ccc} -(I⊗Q)-(I⊗R) & 0 & 0\\ {}* & -(I⊗W_{1i}) & 0\\ {}* & * & -\alpha I \end{array}\right]. \end{array}$$

Hence, we can verify that when $\Xi_{i}\left(h\right)<0$ in Theorem 1 holds, it satisfies that

$$\begin{array}{cc} E\{V(i,t)\} & <e^{at}\eta^{T}\left(0\right)\left(I⊗P_{i}\right)\eta \left(0\right)\\ \; & +e^{at}\int_{-\bar{h}}^{0}\eta^{T}\left(s\right)\left(I⊗Q\right)\eta \left(s\right)ds\\ \; & +e^{at}\bar{h}\int_{-\bar{h}}^{0}\int_{t+\varsigma }^{t}{\dot{\eta }}^{T}\left(s\right)\left(I⊗R\right)\dot{\eta }\left(s\right)dsd\varsigma \\ \; & +ae^{at}\int_{0}^{t}e^{-as}\omega^{T}\left(s\right)\omega \left(s\right)ds,\\ \leq & e^{aT_{F}}(\lambda_{\max}\left\{P_{i}\right\}c_{1}+e^{aT_{F}}\bar{h}(\lambda_{\max}\left\{Q\right\}c_{1}\\ \; & +\frac{1}{2}{\bar{h}}^{3}\lambda_{\max}\left\{R\right\}c_{1}+\bar{\omega }\left(1-e^{-aT_{F}}\right),\\ 0 & \leq t\leq T_{F} \end{array}$$

where $P_{i}=\Lambda^{-1/2}\left(I⊗P_{i}\right)\Lambda^{-1/2},Q=\Lambda^{-1/2}\left(I⊗Q\right)\Lambda^{-1/2},R=\Lambda^{-1/2}\left(I⊗R\right)\Lambda^{-1/2}.$

Furthermore, it can be derived that

$$\begin{array}{cc} E\{V(i,t)\}\geq & E\{\eta^{T}\left(t\right)\left(I⊗P_{i}\right)\eta \left(t\right)\}\\ \geq & \lambda_{\min}\left\{P_{i}\right\}E\left\{\eta^{T}\left(t\right)\Lambda \eta \left(t\right)\right\}. \end{array}$$

Consequently, we have that

$$\begin{array}{cc} \phi_{4i}E\left\{\eta^{T}\left(t\right)\Lambda \eta \left(t\right)\right\}\leq & e^{aT_{F}}[c_{1}\phi_{1i}+\bar{h}c_{1}\phi_{2}+\\ \; & \frac{1}{2}{\bar{h}}^{3}c_{1}\phi_{3}+\bar{\omega }\left(1-e^{-aT_{F}}\right)], \end{array}$$

where

$$\begin{array}{cc} \phi_{1i}= & \lambda_{\max}\left\{P_{i}\right\},\\ \phi_{2}= & \lambda_{\max}\left\{Q\right\},\\ \phi_{3}= & \lambda_{\max}\left\{R\right\},\\ \phi_{4i}= & \lambda_{\min}\left\{P_{i}\right\}. \end{array}$$

This means that η(t) can be finite-time boundedness according to Definition 2, and we thus finish this proof. □

**Theorem** **2.** *For given $\bar{h}$, the finite-time formation problem of UAVs (3) can be solved, if there exist mode-dependent matrices ${\tilde{P}}_{i}≻0$, $\tilde{Q}≻0$, $\tilde{R}≻0$ and constant χ>0, such that $\chi \Lambda^{-1}≺{\tilde{P}}_{i}≺\Lambda^{-1}$, $0≺\tilde{Q}≺2\Lambda^{-1}$, $0≺\tilde{R}≺2\Lambda^{-1}$ and $\Theta_{i,\iota }≺0$ holds for all i∈S and ι∈L, where*

$$\begin{array}{cc} \; & \Theta_{i,\iota }=\left[\begin{array}{cc} \Theta_{1i,\iota } & \Theta_{2i,\iota }\\ {}* & \Theta_{3i,\iota } \end{array}\right],\\ \; & \Theta_{1i,\iota }=\left[\begin{array}{ccc} \Theta_{11i} & \Theta_{12i} & 0\\ {}* & \Theta_{13i} & \left(I⊗\tilde{R}\right)\\ {}* & * & -\left(I⊗\tilde{Q}\right)-\left(I⊗\tilde{R}\right) \end{array}\right],\\ \; & \Theta_{11i}=2\left(I⊗A{\tilde{P}}_{i}\right)+\left(I⊗\tilde{Q}\right)-\left(I⊗\tilde{R}\right)\\ \; & -\alpha \left(I⊗{\tilde{P}}_{i}\right)+\pi_{ii,\iota }\left(I⊗{\tilde{P}}_{i}\right),\\ \; & \Theta_{12i}=\left({\mathrm{EM}}_{i}⊗B{\tilde{K}}_{i}\right)+\left(I⊗\tilde{R}\right), \end{array}$$

$$\begin{array}{cc} \; & \Theta_{13i}=-2\left(I⊗\tilde{R}\right)+\kappa_{i}\left(EE^{T}⊗{\tilde{W}}_{2i}\right)\\ \; & \Theta_{2i}=\left[\Theta_{21i},\Theta_{22i}\right],\\ \; & \Theta_{21i}=\left[\begin{array}{ccc} -({\mathrm{EM}}_{i}⊗B{\tilde{K}}_{i}) & I⊗B_{w} & \bar{h}\left(I⊗A_{i}^{T}{\tilde{P}}_{i}\right)\\ 0 & 0 & \bar{h}\left(M_{i}^{T}E^{T}⊗B^{T}{\tilde{K}}_{i}\right)\\ 0 & 0 & 0 \end{array}\right],\\ \; & \Theta_{22i}=\left[\begin{array}{cccc} \sqrt{\pi_{i,\iota 1}}{\tilde{P}}_{i} & \sqrt{\pi_{i,\iota 2}}{\tilde{P}}_{i}\; & \cdots & \sqrt{\pi_{i,\iota N}}{\tilde{P}}_{i}\\ 0 & 0 & \cdots & 0\\ 0 & 0 & \cdots & 0 \end{array}\right],\\ \; & \Theta_{3i}=\left[\begin{array}{cc} \Theta_{31i}\left(h\right) & \Theta_{32i}\left(h\right)\\ {}* & \Theta_{33i}\left(h\right) \end{array}\right], \end{array}$$

$$\begin{array}{cc} \; & \Theta_{31i}=\left[\begin{array}{ccc} -\gamma {\tilde{P}}_{i} & \bar{h}\left(I⊗B_{w}^{T}\right) & 0\\ {}* & \left(I⊗R\right)-2\left(I⊗{\tilde{P}}_{i}\right) & 0\\ {}* & * & -(I⊗{\tilde{P}}_{1}) \end{array}\right],\\ \; & \Theta_{32i}=\left[\begin{array}{ccc} 0 & \cdots & 0\\ \vdots & \vdots & \vdots \\ 0 & \cdots & 0 \end{array}\right],\\ \; & \Theta_{33i}=\left[\begin{array}{ccc} -(I⊗{\tilde{P}}_{2}) & \cdots & 0\\ {}* & \ddots & 0\\ {}* & * & -(I⊗{\tilde{P}}_{N}) \end{array}\right]. \end{array}$$

*with*

$$c_{1}\left(\frac{1}{\chi }+\frac{2\bar{h}}{\chi^{2}}+\frac{1{\bar{h}}^{3}}{\chi^{2}}\right)+\bar{\omega }\left(1-e^{-aT_{F}}\right)\leq c_{2}e^{-aT_{F}}.$$

**Proof.**  Following similar proofs in Theorem 1, it can be derived that

$$\begin{array}{cc} \; & LV\left(i,t\right)-\alpha \eta^{T}\left(t\right)\left(I⊗P_{i}\right)\eta \left(t\right)\\ \; & -2\eta^{T}\left(t\right)\omega \left(t\right)-\gamma \omega^{T}\left(t\right)\omega \left(t\right)\\ \leq & LV\left(i,t\right)-\alpha \eta^{T}\left(t\right)\left(I⊗P_{i}\right)\eta \left(t\right)-2\eta^{T}\left(t\right)\omega \left(t\right)\\ \; & -\gamma \omega^{T}\left(t\right)\omega \left(t\right)-\epsilon^{T}\left(lh_{i}\right)\left(I⊗W_{1i}\right)\epsilon \left(lh_{i}\right)\\ \; & +\kappa_{i}\eta^{T}\left(t-\tau_{i}\left(t\right)\right)\left(E^{T}E⊗W_{2i}\right)\eta \left(t-\tau_{i}\left(t\right)\right), \end{array}$$

such that ${\tilde{\Theta }}_{i}\left(h\right)<0$ can guarantee that the above matrix inequality holds, where

$$\begin{array}{cc} \; & {\tilde{\Theta }}_{i}=\left[\begin{array}{cc} {\tilde{\Theta }}_{1i} & {\tilde{\Theta }}_{2i}\\ {}* & {\tilde{\Theta }}_{3i} \end{array}\right],\\ \; & {\tilde{\Theta }}_{1i}=\left[\begin{array}{ccc} {\tilde{\Theta }}_{11i} & {\tilde{\Theta }}_{12i} & 0\\ {}* & {\tilde{\Theta }}_{13i} & \left(I⊗R\right)\\ {}* & * & -(I⊗Q)-(I⊗R) \end{array}\right],\\ \; & {\tilde{\Theta }}_{11i}=2\left(I⊗P_{i}A\right)+\left(I⊗Q\right)-\left(I⊗R\right)\\ \; & -\alpha \left(I⊗P_{i}\right)+\pi_{ij}\left(h\right)\left(I⊗P_{i}\right),\\ \; & {\tilde{\Theta }}_{12i}=\left({\mathrm{EM}}_{i}⊗P_{i}BK_{i}\right)+\left(I⊗R\right),\\ \; & {\tilde{\Theta }}_{13i}=-2\left(I⊗R\right)+\kappa_{i}\left(E^{T}E⊗W_{2i}\right)\\ \; & {\tilde{\Theta }}_{2i}=\left[{\tilde{\Theta }}_{21i},{\tilde{\Theta }}_{22i}\right], \end{array}$$

$$\begin{array}{cc} \; & {\tilde{\Theta }}_{21i}=\left[\begin{array}{ccc} -({\mathrm{EM}}_{i}⊗P_{i}BK_{i}) & I⊗P_{i}B_{w} & \bar{h}{\left(I⊗A_{i}\right)}^{T}\\ 0 & 0 & \bar{h}{\left({\mathrm{EM}}_{i}⊗BK_{i}\right)}^{T}\\ 0 & 0 & 0 \end{array}\right],\\ \; & {\tilde{\Theta }}_{22i}=\left[\begin{array}{cccc} \sqrt{\pi_{i1}\left(h\right)}I & \sqrt{\pi_{i2}\left(h\right)}I & \cdots & \sqrt{\pi_{iM}\left(h\right)}I\\ 0 & 0 & \cdots & 0\\ 0 & 0 & \cdots & 0 \end{array}\right],\\ \; & {\tilde{\Theta }}_{3i}=\left[\begin{array}{cc} {\tilde{\Theta }}_{31i} & {\tilde{\Theta }}_{32i}\\ {}* & {\tilde{\Theta }}_{33i} \end{array}\right], \end{array}$$

$$\begin{array}{cc} \; & {\tilde{\Theta }}_{31i}=\left[\begin{array}{ccc} -\gamma I & \bar{h}{\left(I⊗B_{w}\right)}^{T} & 0\\ {}* & -{\left(I⊗R\right)}^{-1} & 0\\ {}* & * & -(I⊗P_{1}^{-1}) \end{array}\right],\\ \; & {\tilde{\Theta }}_{32i}=\left[\begin{array}{ccc} 0 & \cdots & 0\\ \vdots & \vdots & \vdots \\ 0 & \cdots & 0 \end{array}\right],\\ \; & {\tilde{\Theta }}_{33i}=\left[\begin{array}{ccc} -(I⊗P_{2}^{-1}) & \cdots & 0\\ {}* & \ddots & 0\\ {}* & * & -(I⊗P_{N}^{-1}) \end{array}\right]. \end{array}$$

Consequently, it yields that

$$\begin{array}{cc} E\{e^{-aT_{F}}V\left(i,t\right)\}< & 2E\{\int_{0}^{T_{F}}e^{-at}\eta^{T}\left(s\right)\omega \left(s\right)ds\}\\ \; & +\gamma \int_{0}^{T_{F}}e^{-at}\omega^{T}\left(s\right)\omega \left(s\right)ds, \end{array}$$

which holds with E{V(i,t)}>0 that

$$2E\left\{\int_{0}^{T_{F}}\eta^{T}\left(s\right)\omega \left(s\right)ds\right\}>-\tilde{\gamma }\int_{0}^{T_{F}}\omega^{T}\left(s\right)\omega \left(s\right)ds,$$

with $\tilde{\gamma }=\gamma e^{-aT_{F}}.$

Subsequently, by performing matrix congruent transformation to ${\tilde{\Theta }}_{i}<0$ with $I⊗P_{i}^{-1}$ and letting $P_{i}^{-1}={\tilde{P}}_{i},$$P_{i}^{-1}QP_{i}^{-1}=\tilde{Q},$$P_{i}^{-1}RP_{i}^{-1}=\tilde{R},$$K_{i}P_{i}^{-1}={\tilde{K}}_{i},$$P_{i}^{-1}W_{1i}P_{i}^{-1}={\tilde{W}}_{1i},$ $P_{i}^{-1}W_{2i}P_{i}^{-1}={\tilde{W}}_{2i},$ one has

$$\begin{array}{cc} \; & {\hat{\Theta }}_{i}=\left[\begin{array}{cc} {\hat{\Theta }}_{1i} & {\hat{\Theta }}_{2i}\\ {}* & {\hat{\Theta }}_{3i} \end{array}\right],\\ \; & {\hat{\Theta }}_{1i}=\left[\begin{array}{ccc} {\hat{\Theta }}_{11i} & {\hat{\Theta }}_{12i} & 0\\ {}* & {\hat{\Theta }}_{13i}\left(h\right) & \left(I⊗\tilde{R}\right)\\ {}* & * & -\left(I⊗\tilde{Q}\right)-\left(I⊗\tilde{R}\right) \end{array}\right],\\ \; & {\hat{\Theta }}_{11i}=2\left(I⊗A{\tilde{P}}_{i}\right)+\left(I⊗\tilde{Q}\right)-\left(I⊗\tilde{R}\right)\\ \; & -\alpha \left(I⊗{\tilde{P}}_{i}\right)+\pi_{ij}\left(h\right)\left(I⊗{\tilde{P}}_{i}\right),\\ \; & {\hat{\Theta }}_{12i}=\left({\mathrm{EM}}_{i}⊗B{\tilde{K}}_{i}\right)+\left(I⊗\tilde{R}\right),\\ \; & {\hat{\Theta }}_{13i}=-2\left(I⊗\tilde{R}\right)+\kappa_{i}\left(EE^{T}⊗{\tilde{W}}_{2i}\right)\\ \; & {\hat{\Theta }}_{2i}=\left[{\hat{\Theta }}_{21i},{\hat{\Theta }}_{22i}\right], \end{array}$$

$$\begin{array}{cc} \; & {\hat{\Theta }}_{21i}=\left[\begin{array}{ccc} -({\mathrm{EM}}_{i}⊗B{\tilde{K}}_{i}) & I⊗B_{w} & \bar{h}\left(I⊗A_{i}^{T}{\tilde{P}}_{i}\right)\\ 0 & 0 & \bar{h}\left(M_{i}^{T}E^{T}⊗B^{T}{\tilde{K}}_{i}\right)\\ 0 & 0 & 0 \end{array}\right],\\ \; & {\hat{\Theta }}_{22i}=\left[\begin{array}{cccc} \sqrt{\pi_{i1}\left(h\right)}{\tilde{P}}_{i} & \sqrt{\pi_{i2}\left(h\right)}{\tilde{P}}_{i} & \cdots \; & \sqrt{\pi_{iM}\left(h\right)}{\tilde{P}}_{i}\\ 0 & 0 & \cdots & 0\\ 0 & 0 & \cdots & 0 \end{array}\right],\\ \; & {\hat{\Theta }}_{3i}=\left[\begin{array}{cc} {\hat{\Theta }}_{31i} & {\hat{\Theta }}_{32i}\left(h\right)\\ {}* & {\hat{\Theta }}_{33i}\left(h\right) \end{array}\right], \end{array}$$

$$\begin{array}{cc} \; & {\hat{\Theta }}_{31i}=\left[\begin{array}{ccc} -\gamma {\tilde{P}}_{i} & \bar{h}\left(I⊗B_{w}^{T}\right) & 0\\ {}* & \left(I⊗R\right)-2\left(I⊗{\tilde{P}}_{i}\right) & 0\\ {}* & * & -(I⊗{\tilde{P}}_{1}) \end{array}\right],\\ \; & {\hat{\Theta }}_{32i}=\left[\begin{array}{ccc} 0 & \cdots & 0\\ \vdots & \vdots & \vdots \\ 0 & \cdots & 0 \end{array}\right],\\ \; & {\hat{\Theta }}_{33i}=\left[\begin{array}{ccc} -(I⊗{\tilde{P}}_{2}) & \cdots & 0\\ {}* & \ddots & 0\\ {}* & * & -(I⊗{\tilde{P}}_{N}) \end{array}\right]. \end{array}$$

Meanwhile, by letting ${\bar{P}}_{i}=\Lambda^{1/2}{\tilde{P}}_{i}\Lambda^{1/2}$ with

$$\lambda_{\max}\left\{{\bar{P}}_{i}\right\}=\lambda_{\min}\left\{P_{i}\right\},$$

one has

$$I<P_{i}<\frac{1}{\chi }I$$

which leads to $\phi_{1}<\frac{1}{\chi }$ and $\phi_{4}>1$. Moreover, it follows that $\Lambda^{-1/2}Q\Lambda^{-1/2}<$$2{\left(\Lambda^{-1/2}P_{i}\Lambda^{-1/2}\right)}^{2}$$<\frac{2}{\chi^{2}}I$ and $\Lambda^{-1/2}R\Lambda^{-1/2}<2{\left(\Lambda^{-1/2}P_{i}\Lambda^{-1/2}\right)}^{2}<\frac{2}{\chi^{2}}I,$ such that $\phi_{2}<\frac{2}{\chi^{2}}$ and $\phi_{3}<\frac{2}{\chi^{2}}$. Then, we have

$$c_{1}\left(\frac{1}{\chi }+\frac{2\bar{h}}{\chi^{2}}+\frac{1{\bar{h}}^{3}}{\chi^{2}}\right)+\bar{\omega }\left(1-e^{-aT_{F}}\right)\leq c_{2}e^{-aT_{F}}.$$

Finally, by adopting the method for the time-varying dwell time $\pi_{ij}\left(h\right)$, $\pi_{ij}\left(h\right)=\sum_{1}^{L}\lambda_{\iota }\pi_{ij,\iota }$, $\sum_{1}^{L}\lambda_{\iota }=1$, $\lambda_{\iota }\geq 0$, the remainder of the proof can follow conveniently from Theorem 1, which ensures that the finite-time passivity formation can be satisfied according to Definition 3. □

**Remark** **3.** *The computation complexity for the above linear matrix inequality (LMI) convex optimization conditions is related to the convex combination representation of time-varying dwell time $\pi_{ij}\left(h\right)$ with ι∈L. As such, it is a trade-off to setting an appropriate ι for better describing the time-varying dwell time and computation complexity of LMIs.*

## 4. Simulation Results

This section demonstrates the effectiveness of theoretical results via the numerical simulation results.

**Figure 1:**
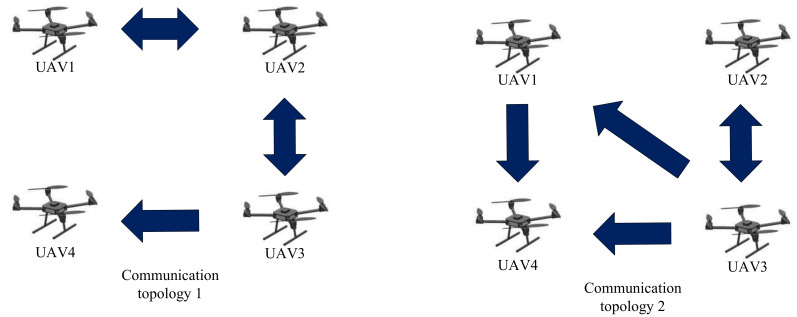
Illustration of switching communication topology of UAVs.

As depicted in Figure 1, consider a group of four UAVs and the corresponding Laplacian matrices of communication topology are given by

$$\begin{array}{cc} L_{1}= & \left[\begin{array}{cccc} 1 & -1 & 0 & 0\\ -1 & 2 & -1 & 0\\ 0 & -1 & 1 & 0\\ 0 & 0 & -1 & 1 \end{array}\right],\\ L_{2}= & \left[\begin{array}{cccc} 1 & 0 & -1 & 0\\ 0 & 1 & -1 & 0\\ 0 & -1 & 1 & 0\\ -1 & 0 & -1 & 2 \end{array}\right], \end{array}$$

with

$$\begin{array}{cc} M_{1}= & \left[\begin{array}{ccc} 1 & 0 & 0\\ -1 & 1 & 0\\ 0 & -1 & 0\\ 0 & 0 & -1 \end{array}\right],\\ M_{2}= & \left[\begin{array}{ccc} 1 & 1 & 0\\ 0 & 1 & 0\\ 0 & -1 & 1\\ -1 & -1 & -2 \end{array}\right]. \end{array}$$

The transition rates of semi-Markov topologies are assumed to be $\pi_{11}\left(h\right)\in \left[-1.6,-1.4\right]$ and $\pi_{22}\left(h\right)\in \left[-1.7,-1.3\right]$. Accordingly, we can set ι=2 and it follows that $\pi_{11,1}=-1.4$, $\pi_{11,2}=-1.6$, $\pi_{22,1}=-1.3$ and $\pi_{22,2}=-1.7$. The formation configuration is supposed to be $d_{12}={\left[-10,0\right]}^{T}$, $d_{23}={\left[0,-10\right]}^{T}$, $d_{24}={\left[10,-10\right]}^{T}$, $d_{34}={\left[10,0\right]}^{T}$. For the event-triggered communication, the scalar parameters are set by $\kappa_{1}=0.2$ and $\kappa_{2}=0.3$ with $h_{1}=0.05$ s and $h_{2}=0.1$ s. In terms of Theorem 2 with finite-time passivity performance $\left(2500,10^{14},100,2.0612,5\right)$, the values of mode-dependent formation controller gains and triggering matrices are achieved by solving the convex optimization problem as follows:

$$\begin{array}{cc} K_{1}= & \left[\begin{array}{cc} -0.0660 & -0.2413 \end{array}\right],\\ K_{2}= & \left[\begin{array}{cc} 0.0118 & -0.4973 \end{array}\right], \end{array}$$

and

$$\begin{array}{cc} W_{11}= & \left[\begin{array}{cc} 1.2454 & 0.3238\\ 0.3238 & 1.5785 \end{array}\right], \end{array}$$

$$\begin{array}{cc} W_{12}= & \left[\begin{array}{cc} 4.4888 & 0.3403\\ 0.3403 & 5.1241 \end{array}\right],\\ W_{21}= & \left[\begin{array}{cc} 0.0892 & 0.0239\\ 0.0239 & 0.1816 \end{array}\right],\\ W_{22}= & \left[\begin{array}{cc} 0.0796 & 0.0072\\ 0.0072 & 0.2207 \end{array}\right]. \end{array}$$

**Figure 2:**
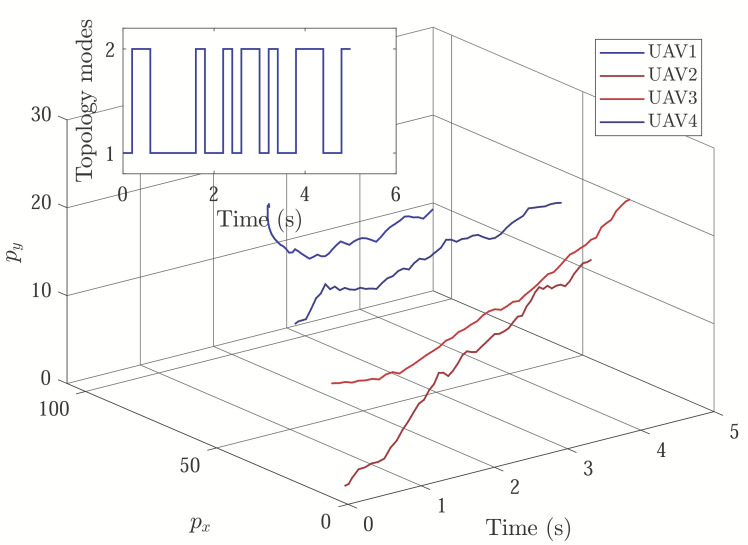
Formation state trajectories of UAVs.

**Figure 3:**
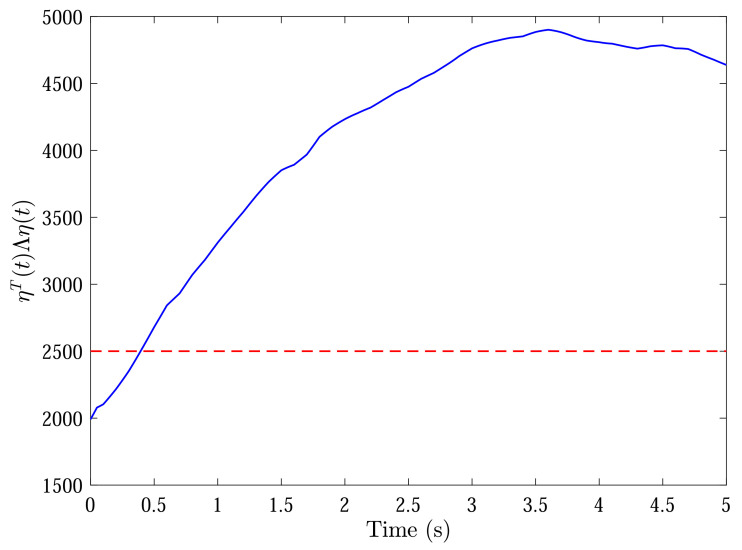
State trajectories of $\eta^{T}\left(t\right)\Lambda \eta \left(t\right)$ (bule) with $c_{1}$ (red).

**Figure 4:**
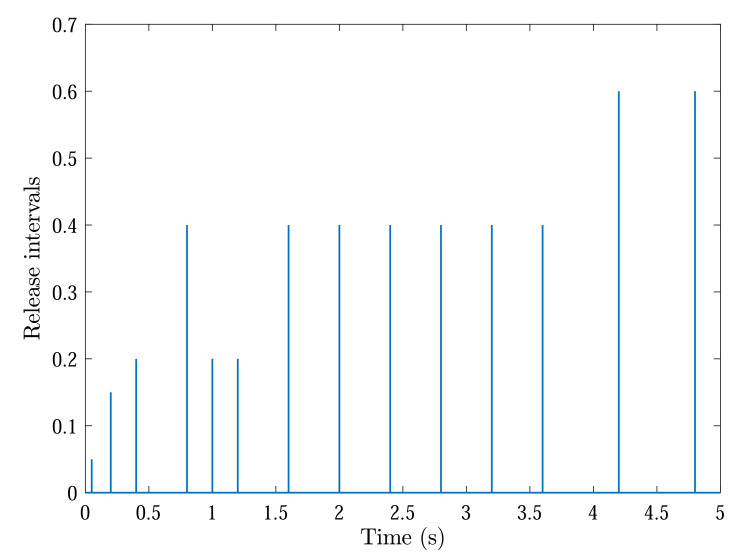
Event-triggered communication instants and release intervals of UAV 1.

**Figure 5:**
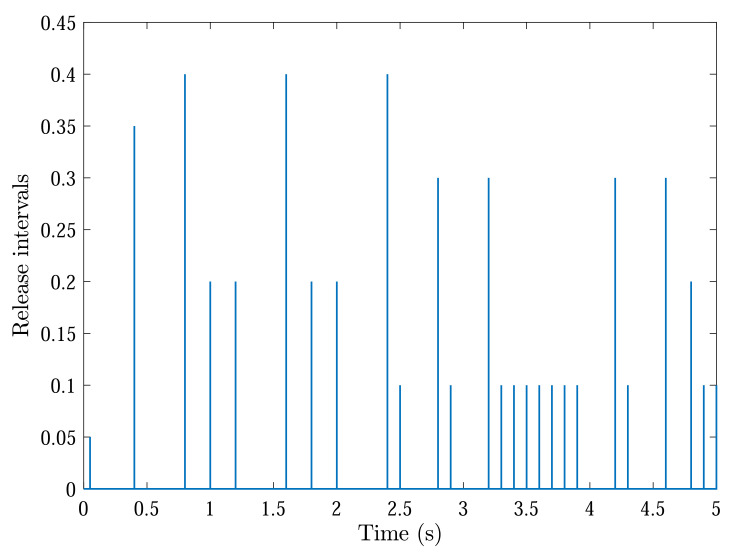
Event-triggered communication instants and release intervals of UAV 2.

**Figure 6:**
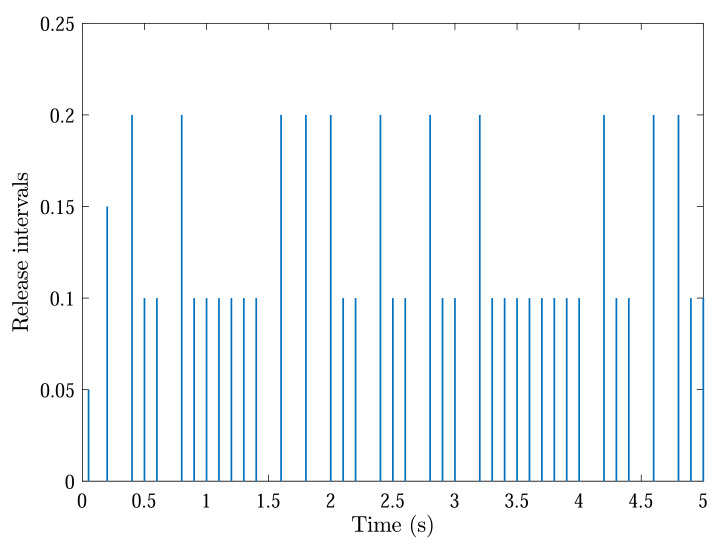
Event-triggered communication instants and release intervals of UAV 3.

**Figure 7:**
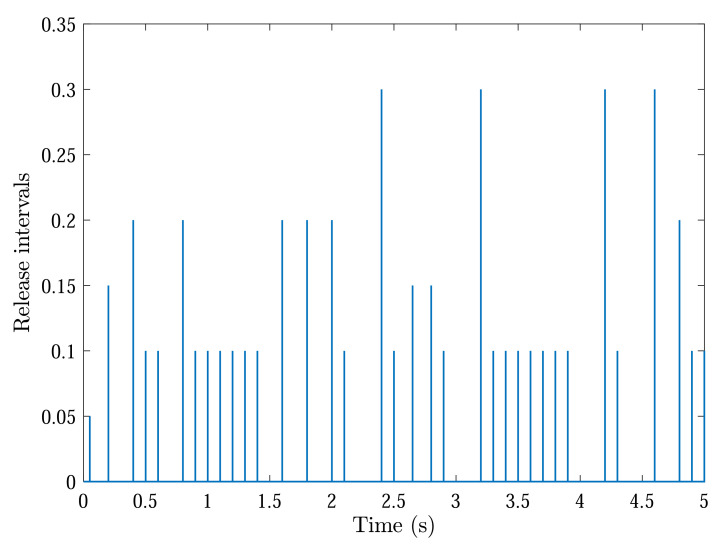
Event-triggered communication instants and release intervals of UAV 4.

**Figure 8:**
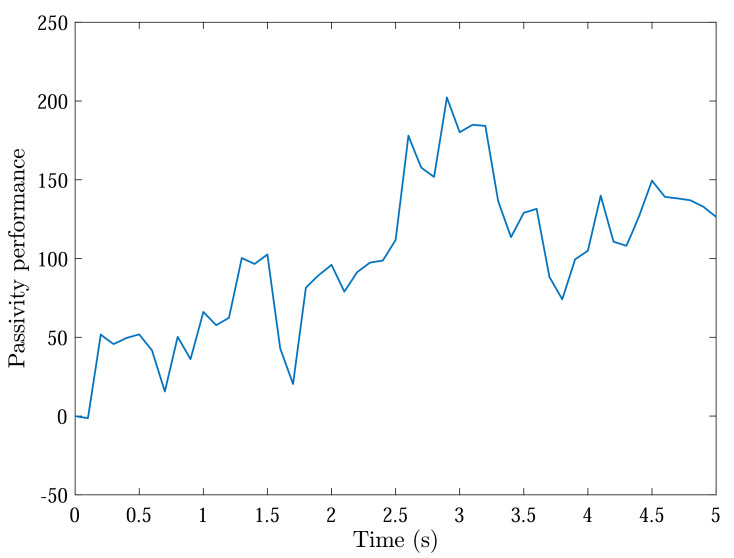
Finite-time passivity performance.

With initial conditions and setting $t_{F}=5$, Figure 2 and Figure 3 show the resulting formation trajectories of UAVs. More precisely, it can be observed that the UAVs can achieve converging motion within the prescribed finite time interval. Moreover, one can verify from Figure 3 that the finite-time boundedness can be ensured with $c_{1}$ and $c_{2}$ by Definition 2. Figure 4, Figure 5, Figure 6 and Figure 7 reveal the data broadcasting instants with release intervals of UAVs, from which one can find that the signal transmissions can be effectively decreased compared with the traditional time-triggered approaches, such that the local communication efficiency among the UAVs can be increased under the distributed formation control framework. Furthermore, it can be seen from Figure 8 that the finite-time passivity condition can be satisfied according to Definition 3 with

$$\begin{array}{c} 2E\left\{\int_{0}^{5}\eta^{T}\left(s\right)\omega \left(s\right)ds\right\}+\gamma \int_{0}^{5}\omega^{T}\left(s\right)\omega \left(s\right)ds\geq 0. \end{array}$$

## 5. Conclusions

This paper studies the formation problem of UAVs with semi-Markov jump topologies under the finite-time passivity performance. By proposing an asynchronous event-triggered data transmission strategy, communication efficiency can be improved considerably. With the aid of choosing the mode-dependent Lyapunov–Krasovskii function, sufficient formation criterion is established, and corresponding formation controller gains are calculated by LMIs, such that desired finite-time passivity performance is satisfied for configuration formation. A simulation example with four UAV verifications is performed to validate the effectiveness of the derived theoretical algorithm. Future research of interest can be focused on the cases with more complex network environments, i.e., limited bandwidth or channel fading of communication network.

## Figures and Tables

**Table 1:** Notations.

Symbol	Implication
$R^{n}$	*n* dimensional Euclidean space matrices
A≻0	Positive symmetric definite matrix A
(O,F,P)	Complete probability space
Pr{·}	Probability
A⊗B	Kronecker product
*	Symmetry term in matrix
E{·}	Mathematics expectation of a stochastic process

**Table 2:** Notation of event triggering parameters.

Symbol	Implication
$t_{k}^{m}$	Latest *k*th updating instant of the *m*th UAV
$\kappa_{\delta (t)}$	Triggering threshold parameter
$W_{1\delta (t)},W_{2\delta (t)}$	Triggering weighting matrices
$\chi_{m}\left(t_{k}^{m}h_{\delta (t)}+l_{m}h_{\delta (t)}\right)$	Single UAV state changes
$\varkappa_{m}\left(t_{k}^{m}h_{\delta (t)}+l_{m}h_{\delta (t)}\right)$	Local neighboring UAVs state changes

## Data Availability

All data is within this paper.
